# Re-examining the social gradient in health: A study of Dutch men, 1850–1984

**DOI:** 10.1016/j.ssmph.2023.101518

**Published:** 2023-09-21

**Authors:** Kristina Thompson, Johan van Ophem

**Affiliations:** a Health and Society, Social Sciences Department, Wageningen University & Research, the Netherlands; b Urban Economics, Social Sciences Department, Wageningen University & Research, the Netherlands

## Abstract

Today, a social gradient in health is clearly visible. Individuals with higher socio-economic statuses tend to live longer lives, and are less likely to be disabled or chronically ill. However, there is debate over when the social gradient emerged: is it a constant across contexts, or a particular feature of certain societies? Often, social gradients are not found in historical contexts. This is perhaps because historical studies use mortality as their sole measure of health, which may not fully reflect the health statuses of the living.

Using another health indicator may help to identify whether a social gradient in health was present in historical contexts. One alternative measure of health is body height, a barometer of population health. In this study, we accordingly examined socio-economic status’s relationship to both adult mortality and body height. A sample of Dutch men (n=3396), born between 1850 and 1900, was used. Socio-economic status was measured with parental occupational class, and height was measured at age 20. Survival analyses (for mortality at age 20 or later) and linear regressions (for height at age 20) were performed.

We found no clear gradient in occupational class’s relationship to adult mortality. Regarding height, individuals from elite backgrounds were estimated to be 2.82 cm cm taller (95% CI: 1.41–4.24) than those from unskilled working backgrounds. While a gradient in height was present in earlier birth cohorts, it was not visible among men born between 1885 and 1900.

These findings indicate that there was a social gradient in health in the late nineteenth and twentieth centuries, although the gradient perhaps changed based on the indicator and time period being examined. This may mean that the social gradient in health is more persistent over time than it appears when only examining the social gradient in mortality.

## Highlights

• A clear gradient in socio-economic status and body height was found.
• This indicates that there was a social gradient in health in the late nineteenth and twentieth centuries.
• There was no gradient found between socio-economic status and age at death.
• However, in some analyses, farmers had a longevity advantage.
• This suggests that disease exposure was more important than health status to mortality.

## 1 Introduction

Today, the poorest people have shorter life expectancies and more diseases and disabilities than the richest within a given country (Marmot, 2003). This generally translates to between five and ten years of shorter life expectancy at birth, and between ten and twenty years fewer years of disability-free living (Mackenbach, 2012). In contemporary populations, this positive gradient has been found between various indicators of both socio-economic status (SES) and health (Wolfe et al., 2012).

However, there is not yet agreement whether the social gradient in health is a constant feature of societies, or whether it is a product of a specific set of circumstances present in contemporary societies. In the former instance, Link and Phelan (1995) argued that socio-economic status is a ‘fundamental cause’ of health, which underlies more proximal risk factors and persists through time. The risk factors themselves may change. For instance, differences in infectious disease risks may give way to different lifestyle practices, such as diet, smoking and exercise. However, the underlying relationship between socio-economic status and health persists, because it is caused by differential access to resources, and occurs through multiple pathways (Link & Phelan, 1995).

In contrast, Antonovsky (1967) argued that the social gradient depends on contextual factors that change over time. He identified four major periods in Europe: (1) prior to 1650, when there were no differences in health based on SES; (2) from 1650 to 1850, when an increasing gap in life expectancy emerged, either because the poor became less healthy, or because the wealthy became healthier; (3) from 1850 to 1930, in which life expectancy increased again, largely driven by improvements in health among those from lower socio-economic backgrounds; and (4) the mid-twentieth century onward, in which the eradication of many infectious diseases improved life expectancy for all. This has become known as the divergence-convergence hypothesis.

Recently, Link, Phelan and colleagues have adapted the fundamental cause theory. The authors still argued that social inequalities result in inequalities in health, and that this is a constant feature across societies (Clouston et al., 2016). However, how social inequalities in disease translate to inequalities in mortality depends heavily on the context (Clouston et al., 2016). In other words, a social gradient in mortality is not necessarily a constant feature of society, although a social gradient in health overall may be.

A number of studies have empirically examined whether a social gradient in health was present in historical populations, and have arrived at conflicting conclusions (for an overview, see Bengtsson & van Poppel, 2011). In Sweden, Bengtsson et al. (2020) found that a social gradient in adult mortality only emerged around 1950 for women, and around 1970 for men. Using a similar dataset to this study’s, Schenk & van Poppel (2011) found no evidence of class differences in mortality for Dutch men and women, birth years 1850–1922.

Other studies have found health differences in historical settings (Jaadla et al., 2017). Schumacher and Oris (2011) found persistent evidence of a social gradient in mortality in Geneva, from the seventeenth century onward. However, they found that the social gradient was driven by infant and child mortality prior to the twentieth century, and by adult mortality in the twentieth century (Schumacher & Oris, 2011). Similarly, studies in Sweden and the Netherlands have found evidence of social gradients in infant and child mortality prior to the twentieth century (Dribe & Karlsson, 2022; van Poppel et al., 2005).

This debate may in part stem from studies’ measure of health. These studies were often interested in understanding if a social gradient in *health* was present, but only examined whether a social gradient in *mortality* was present. However, health is much more than the age at which someone dies (Huber et al., 2011). In the contemporary public health literature, the social gradient in health is examined with a wide variety of health indicators (Blázquez et al., 2014; Marmot, 2003; Stronks et al., 1997). Mortality is an extreme indicator of health, and may fail to reflect more moderate gradations in health that are evident in more sensitive measures, such as morbidity and self-rated health (Nolte & McKee, 2003). While mortality is the most widely-available measure of health in historical populations, using mortality as the sole indicator of health may be insufficient to determine whether a social gradient in health overall was present.

In this study, we sought to enrich the understanding of SES’s relationship to health, by exploring SES’s relationship to both adult mortality and another indicator of health. One such indicator is body height, which provides information on population health. The taller populations are, the healthier they tend to be. This is because height partially reflects the quantity and quality of food an individual consumes, minus different claims on their bodies (Baten & Komlos, 1998). These claims may include disease, stress, physical labor, and basal metabolism, among others (Floud et al., 2011). Already, studies have found clear socio-economic gradients in height (Floud et al., 2011; Öberg, 2014). Examining the relationships between SES and both height and adult mortality may help to paint a more complete picture of the social gradient in health.

### 1.1 The Dutch context

We examined these relationships among a sample of men born in the late nineteenth century Netherlands. This period saw a less virulent disease environment, increased industrialization of food production, and public health and sanitation projects (Wolleswinkel-van den Bosch et al., 2000). The Netherlands was also a relatively late industrializer, with industrialization beginning around 1860 (Mokyr, 2000). This meant that the country avoided many of the negative health consequences of industrialization, including those related to unsafe working conditions and child labor (Quanjer, 2023).

These factors likely facilitated a mortality decline. Infant mortality began to decrease first, around 1870 (Wolleswinkel-van den Bosch et al., 2000). This was followed by adults ages 20 to 49 around 1880, and children ages 1 to 14 around 1890 (Wolleswinkel-van den Bosch et al., 2000). Mortality decline further accelerated for all age groups between the period of 1917 and 1922 (Wolleswinkel-van den Bosch et al., 2000). Between 1870 and 1939, average life expectancy in the Netherlands increased from 37 years to 67 years (Wolleswinkel-van den Bosch et al., 2000).

The Dutch were also growing during the late nineteenth and early twentieth centuries, likely spurred by similar factors driving the decrease in mortality (Grasgruber & Hrazdíra, 2020). This in itself was not remarkable. The secular increase in height occurred throughout Europe and North America during this period (Baten & Blum, 2012). However, the pace at which the Dutch experienced the secular trend in body height was unique: the Dutch grew from among the shortest nations in Europe and North America in the early nineteenth century to the tallest in the world by the mid-twentieth (Grasgruber & Hrazdíra, 2020).

## 2 Methods

### 2.1 Data sources and sample construction

Against this backdrop, we studied the life courses of Dutch men. This study used a dataset derived from the Historical Sample of the Netherlands (HSN). The HSN is a ∼0.5% representative sample of the Dutch population, birth years 1812–1922, and contains, at a minimum, birth certificates for these individuals (Mandemakers, 2000). Death certificates, when available, and marriage certificates, when applicable and available, were also included in the HSN. From 1850, population registers, which contains more detailed information on household composition, were implemented. Between 1910 and 1939, this was followed by family cards, which contain information similar to population registers, and are based around the household as the unit of observation (Mandemakers, 2000). From 1939 onward, family cards were replaced with personal cards, so that individuals became the units of observation. Because of the richer information available from 1850, this year is our study’s starting point. The 2010 release of the HSN, the version used in this study and the most recent, contains 37,137 life courses (Mandemakers & Kok, 2020).

Next, a sample of HSN research persons (RPs) was linked to their conscription records in the Heights and Life Courses database. Only men were included in this sample, as women were not conscripted in the Netherlands. This database contains conscription information from nine of the eleven nineteenth-century Dutch provinces (Mandemakers, 2019). Conscription records generally contain height information when men were twenty years old. Although the Heights and Life Courses database includes RPs born until 1922, few RPs in this dataset (n=98) were born after year 1900, with these men more affected by the Spanish Flu and the Second World War during childhood and young adulthood. We therefore only included men born between 1850 and 1900.

**Fig. 1:**
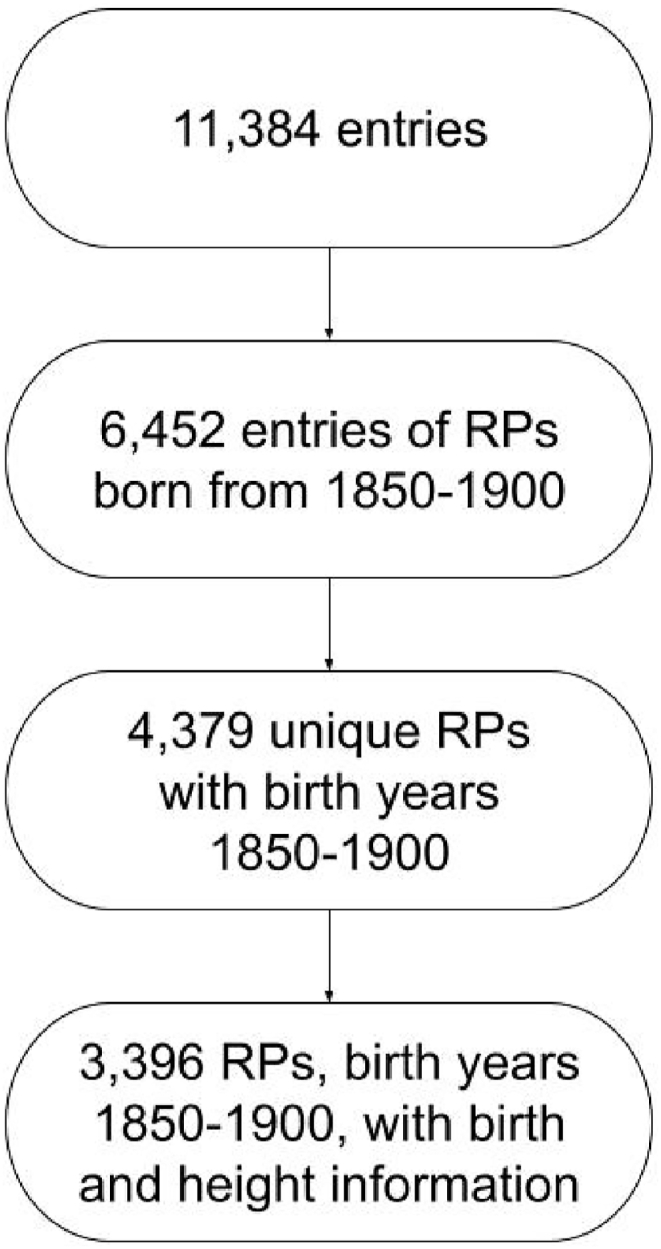
Flowchart of sample selection.

The Heights and Life Courses database contains 11,384 entries from HSN RPs born between 1812 and 1922, and 6,452 entries from individuals born between 1850 and 1900. Of these 6,452 entries, 4,379 were from unique individuals. From there, RPs without birth or height information were excluded. This yielded a sample of 3,396 RPs, with 3,076 RPs also having death information. Fig. 1 presents the sample selection steps.

### 2.2 Variables

In this study, two dependent variables were used: age at death, in years, and body height measured at conscription, in centimeters (cm). The key independent variable was parental occupation on the RP’s birth certificate, to proxy socio-economic status during the RP’s early life. Given that men were generally the breadwinners during this period, we used paternal occupation in cases where both mother and father reported occupational information. However, maternal occupation was used if no paternal occupation was recorded.

Parental occupational status, rather than the RP’s occupational status, was used in our main analyses. The only moment at which all RPs' occupational statuses were measured at similar ages was at conscription, when RPs were 20 years old. Using occupational status at age 20 would be problematic because it increased over men’s working lives. Using the occupation of their parents – particularly of their fathers – helped to contend with this issue. Parental occupational status also reflected the resources available when RPs were growing, something that is a key predictor of body height (Quanjer & Kok, 2019).

To quantify occupational status, the HISCLASS5 scale was used. HISCLASS is a widely-used and validated historical occupational classification system, with its condensed, five-category version most appropriate for inclusion in quantitative analyses of relatively small samples (van Leeuwen & Maas, 2011). Six occupational categories were included in analyses: elite; middle class; skilled workers; farmers; unskilled workers, and having an unknown occupation.

Further, a number of variables known to impact the relationships between occupational status and both height and mortality were included as covariates. Among them was birth year, which was included as a categorical variable in the full-sample analyses, and as a continuous variable in the analyses stratified by birth cohort (adding a quadratic term did not result in a better-fitting model). We also included categorical variables of birth region, the population size of the municipality of birth, derived from the 1889 census (Statistics Netherlands, 2011), and the infant mortality rate in the year and municipality of the RP’s birth as an indicator of the disease environment (Ekamper & van Poppel, 2008). Several factors related to the social practices and resources of the household were also included, namely religion, sibship size when the RP was ten years old, and whether the RP experienced parental death during childhood (up until age 12).

### 2.3 Analyses

Sample characteristics were computed. The relationship between occupational status and mortality was examined first. A Kaplan-Meier curve, stratified by parental occupational status, was generated. Cox proportional hazards regressions were used to examine the relationship between HISCLASS5 score and the hazard of death, or the risk of dying at a given moment in time. Using Cox regressions enabled us to include information on individuals with death information (failures), as well as those without death information (censored cases), which was the case for 320 RPs in this study (Cox, 1972). The relationship between occupational status and body height was examined second. To descriptively examine this relationship, we generated a line graph of height by birth cohort, stratified by parental HISCLASS5 score. Ordinary least squares (OLS) regressions were then used. Both the Cox and OLS regressions were adjusted for the covariates described in section 2.2. The results of analyses using the full sample are presented, as well as results of analyses stratified by birth cohorts.

We performed several additional analyses to better understand our main findings, which are reported in the Appendix. Life tables stratified by parental occupation were computed. To see whether a social gradient in mortality was present at different ages, we used age-stratified mortality analyses, from ages 20 to 60, and from age 60 on. No relationships were found between parental occupational status under age 60, although farmers had a lower hazard of death at age 60 and older.

To assess if our characterization of SES impacted our findings, we replicated the occupational status and age at death analyses using the RP’s highest occupational status. In these analyses, we only included RPs who survived until age 40, as those who died at younger ages tended to have lower occupational statuses. We again found that being a farmer, as well as being an unskilled worker, were associated with a lower hazard of death, relative to being a skilled worker.

Further, we replicated this study’s main analyses using a different measure of occupational status. Rather than the categorical HISCLASS5 classification, we used the continuous HISCAM scale (Lambert et al., 2013). The Netherlands-specific version of the HISCAM scale range from 42 (very low occupation) to 99 (very high occupation), and has been used to study intergenerational mobility in this study’s research period (Thompson & Portrait, 2021). We include a quadratic HISCAM score term in these analyses, because this resulted in a better-fitting model. The results of these analyses align closely with our main analyses.

To interpret this study’s main findings, we tested whether height and mortality were related with Cox regressions. These analyses were stratified by birth cohort, and adjusted for the covariates described in section 2.2. A quadratic height term was also included, as this resulted in a better-fitting model. We found that being taller, up to a certain point of tallness, was associated with a lower risk of death.

## 3 Results

### 3.1 Sample characteristics

Table 1 presents this study’s sample characteristics. Among the 3,076 RPs with death information, the average age of death was 67.5 years (and with a median age at death of 72.35 years). The average height was 168.1 cm. In terms of parental occupational class, 2.9% of RPs had parents with elite occupations, including lawyers and medical doctors. A further 20.5% of RPs had parents with middle class occupations, such as merchants and office workers, and 33.1% had parents who were skilled workers, such as smiths and tailors. Additionally, 19.4% of RPs had parents who were farmers, which could be anyone from a landowner to a farmhand, and 23.2% of RPs had parents who were unskilled workers, such as day laborers or servants. Finally, 0.9% of RPs had no parental occupation listed, either because their parents were unemployed, or because no occupation was recorded.

**Table 1:** Sample characteristics.

	Obs.	%/Mean (SE)
**Age at death (years)**	3,076	67.5
**Height (cm)**	3,396	168.1 (6.9)
**Parental occupational class (HISCLASS)**		
Elite	97	2.9%
Middle class	697	20.5%
Skilled workers	1,124	33.1%
Farmers	659	19.4%
Unskilled workers	789	23.2%
Unknown/no occupation	30	0.9%
**Birth cohort**		
1850–1869	995	29.3%
1870–1884	1,248	36.8%
1885–1900	1,153	34.0%
**Birth region**		
North (Groningen, Friesland, Drenthe)	828	24.4%
Middle (Utrecht, Overijssel, Gelderland)	720	21.2%
Coastal (Noord-Holland, Zuid-Holland)	1,204	35.2%
South (Limburg, Noord-Brabant, Zeeland)	644	19.0%
**Population size of municipality at birth quintile (1889 census)**		
First	283	19.3
Second	256	17.5
Third	315	21.5
Fourth	270	18.4
Fifth	341	23.3
**Infant mortality rate quintile in year of birth**		
First	265	18.1
Second	282	19.3
Third	306	20.9
Fourth	327	22.3
Fifth	278	19.0
Unknown	7	0.5
**Religion**		
Catholic	1,130	33.3%
Liberal Protestant	1,631	48.0%
Neo-Calvinist Protestant	284	8.4%
Jewish	88	2.6%
No/unknown religion	263	7.7%
**Number of siblings at age 10**		
Only child	68	2.0%
One sibling	214	6.3%
2-4 siblings	1,233	36.3%
5-7 siblings	1,280	37.7%
8 or more siblings	601	17.7%
**Experienced parental death before age 12?**		
No	2,620	77.2%
Maternal death	338	10.0%
Paternal death	385	11.3%
Orphan	53	1.6%

### 3.2 SES’s relationship to the age at death

**Fig. 2:**
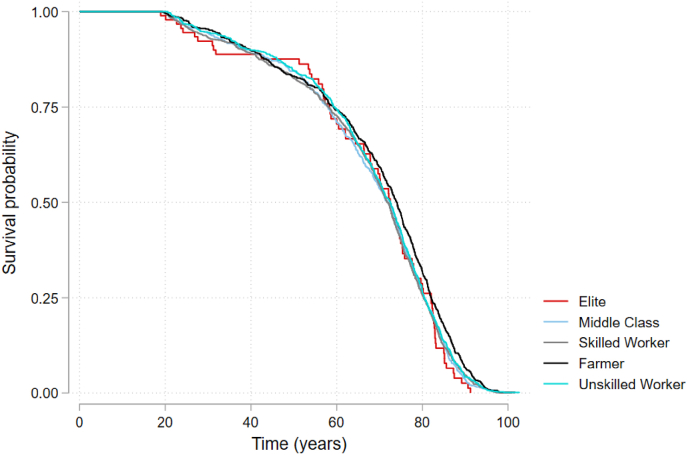
Kaplan Meier curve, stratified by parental occupational status.

Fig. 2 presents a Kaplan Meier curve of RPs’ probability of survival after age 20, stratified by parental occupational class. The overlapping curves indicate little difference in survival probabilities by parental occupational status, and were not significant, based on the results of a log-rank test.

The results of the Cox proportional hazards regressions are presented in Table 2. In all panels, there was no evidence of occupational class-based differences in adult mortality, except among farmers in the full sample panel: being from a farming background was associated with 0.90 times the hazard of death (95% CI: 0.80–1.01), relative to being from a skilled working background.

**Table 2:** Parental occupational status’s relationship to age at death, Cox proportional hazards models.

	1850–1900 (n=3,396; failures/deaths=3,076)			1850–1869 (n=995; failures/deaths=920)			1870–1884 (n=1,248; failures/deaths=1,130)			1885–1900 (n=1,153; failures/deaths=1,026)		
	HR	95% CI		HR	95% CI		HR	95% CI		HR	95% CI	
**Parental occupational status**												
Elite	1.08	0.85	1.37	0.83	0.53	1.31	1.20	0.81	1.76	1.14	0.74	1.73
Middle class	1.02	0.92	1.14	1.03	0.84	1.27	1.04	0.87	1.26	0.97	0.80	1.19
Skilled workers	1.03	0.93	1.14	1.03	0.86	1.23	1.12	0.95	1.33	0.89	0.75	1.07
Farmers	0.90	0.80	1.01	0.95	0.78	1.16	0.89	0.74	1.08	0.87	0.71	1.07
Unskilled workers	Ref.	Ref.	Ref.	Ref.	Ref.	Ref.	Ref.	Ref.	Ref.	Ref.	Ref.	Ref.
Unknown/no occupation	0.92	0.61	1.38	1.22	0.58	2.57	1.25	0.62	2.53	0.56	0.27	1.14
**Birth cohort**												
1850–1869	1.15	1.05	1.26									
1870–1884	Ref.	Ref.	Ref.									
1885–1900	0.92	0.85	1.01									
**Birth year**				0.98	0.97	1.00	0.99	0.98	1.00	0.98	0.96	1.00
**Birth region**												
North	0.85	0.75	0.96	0.89	0.72	1.12	0.82	0.67	1.00	0.86	0.69	1.08
Middle	Ref.	Ref.	Ref.	Ref.	Ref.	Ref.	Ref.	Ref.	Ref.	Ref.	Ref.	Ref.
Coastal	0.90	0.80	1.01	0.91	0.70	1.18	0.82	0.67	1.01	0.98	0.80	1.20
South	0.92	0.82	1.05	0.96	0.76	1.21	0.78	0.63	0.97	1.18	0.94	1.47
Unknown	1.72	0.82	3.62	0.91	0.22	3.72	2.32	0.32	16.83	2.22	0.47	10.40
**Population size in year of birth quintile**												
First	1.00	0.88	1.13	1.06	0.83	1.35	0.97	0.80	1.18	0.99	0.78	1.25
Second	1.09	0.97	1.23	1.19	0.97	1.46	1.07	0.88	1.30	1.13	0.90	1.41
Third	Ref.	Ref.	Ref.	Ref.	Ref.	Ref.	Ref.	Ref.	Ref.	Ref.	Ref.	Ref.
Fourth	1.03	0.91	1.16	0.98	0.78	1.22	0.92	0.74	1.13	1.24	0.99	1.56
Fifth	1.10	0.94	1.29	1.36	0.98	1.91	0.91	0.71	1.18	1.25	0.92	1.69
**Infant mortality rate in year of birth**												
First	1.01	0.89	1.14	0.94	0.73	1.21	0.95	0.76	1.19	1.07	0.85	1.34
Second	1.04	0.92	1.17	0.83	0.64	1.06	1.07	0.86	1.32	1.21	0.97	1.52
Third	Ref.	Ref.	Ref.	Ref.	Ref.	Ref.	Ref.	Ref.	Ref.	Ref.	Ref.	Ref.
Fourth	0.97	0.86	1.10	0.82	0.62	1.08	1.11	0.91	1.36	0.88	0.65	1.18
Fifth	0.97	0.85	1.10	0.82	0.63	1.06	1.09	0.89	1.34	0.85	0.64	1.12
Unknown	0.77	0.38	1.55	8.62	1.17	63.74	1.81	0.11	29.50	0.58	0.21	1.61
**Religion**												
Catholic	0.96	0.87	1.06	1.06	0.88	1.28	1.05	0.89	1.24	0.83	0.70	0.99
Liberal Protestant	Ref.	Ref.	Ref.	Ref.	Ref.	Ref.	Ref.	Ref.	Ref.	Ref.	Ref.	Ref.
Neo-Calvinist	0.94	0.81	1.07	0.88	0.67	1.16	0.84	0.67	1.06	1.07	0.84	1.37
Jewish	2.18	1.70	2.79	1.64	0.97	2.76	2.26	1.52	3.37	2.81	1.86	4.23
No religion	0.90	0.78	1.04	0.85	0.65	1.11	0.87	0.68	1.10	1.03	0.79	1.35
**Number of siblings at age 10**												
Only child	1.06	0.79	1.42	1.32	0.82	2.14	1.07	0.67	1.69	0.92	0.50	1.67
One sibling	1.08	0.93	1.27	1.19	0.91	1.57	1.14	0.89	1.45	0.88	0.64	1.21
2-4 siblings	Ref.	Ref.	Ref.	Ref.	Ref.	Ref.	Ref.	Ref.	Ref.	Ref.	Ref.	Ref.
5-7 siblings	0.99	0.91	1.07	1.13	0.97	1.31	0.96	0.83	1.10	0.91	0.78	1.05
8 or more siblings	0.95	0.85	1.05	1.02	0.83	1.26	0.88	0.73	1.04	1.02	0.86	1.21
**Parental death before age 12?**												
No	Ref.	Ref.	Ref.	Ref.	Ref.	Ref.	Ref.	Ref.	Ref.	Ref.	Ref.	Ref.
Maternal death	0.98	0.87	1.11	0.78	0.63	0.96	1.13	0.92	1.40	1.03	0.83	1.28
Paternal death	1.08	0.96	1.21	1.14	0.93	1.41	0.99	0.83	1.18	1.18	0.95	1.48
Orphan	0.98	0.73	1.32	0.97	0.59	1.61	1.30	0.82	2.07	0.78	0.40	1.52
Likelihood-ratio chi^2^ (p-value reported)	90.74	0.000		42.65	0.063		46.60	0.027		65.44	0.000	

### 3.3 SES’s relationship to body height at conscription

**Fig. 3:**
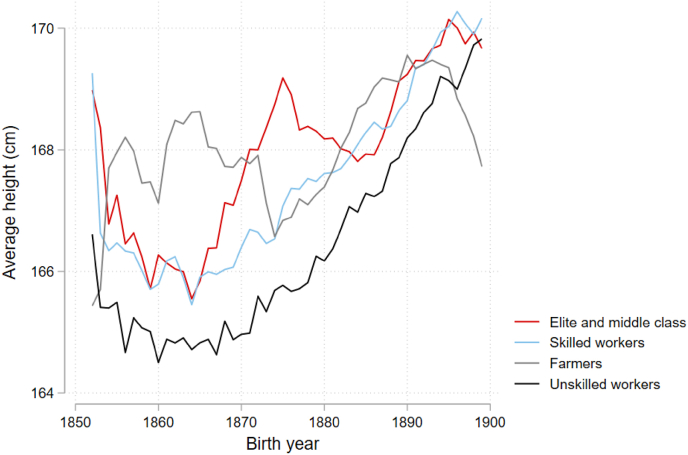
Moving average of height, stratified by parental HISCLASS5 score.

Fig. 3 illustrates the moving average of height by parental HISCLASS5 score. In this figure, RPs from elite and middle class backgrounds are combined into one category, for data sufficiency’s sake. Across all HISCLASS5 categories, there was a general increase in height, and a decrease in class differences in height between 1850 and 1900. Height differences by parental occupational class were most pronounced in 1850: sons of elite and middle class parents had an average height of 168.99 cm, and sons of skilled workers had an average height of 169.26 cm. In contrast, sons of farmers had an average height of 165.43 cm, and sons of unskilled workers had an average height of 164.78 cm. In 1900, differences based on occupational class were smaller. Sons of skilled workers were narrowly the tallest, with an average height of 170.17 cm, followed by sons of unskilled workers (169.82 cm), sons of elite and middle class parents (169.67 cm), and farmers (167.72 cm). The difference between elites and the middle classes and unskilled workers decreased from 4.21 cm in 1850 to −0.15 cm in 1900.

Between 1850 and 1900, there was a remarkable increase in the average heights of unskilled workers, whose average height increased 5.05 cm between 1850 and 1900. Unskilled workers – those who were the shortest at the beginning of the research period – saw the largest increases in average heights over the latter half of the nineteenth century.

Table 3 presents the OLS regression results of parental occupational status’s relationship to height at conscription. Examining the full-sample panel, being from an elite background was associated with a 2.82 cm (95% CI: 1.41–4.24) increase in height at conscription, relative to being from an unskilled worker background. This was followed by being from a middle class background, which was associated with a 1.59 cm (95% CI: 0.90–2.28) increase in height. Next, being from a farming background, and being from a skilled worker background were also associated with being taller, relative to being from an unskilled worker background. In terms of the results stratified by birth cohort, the largest social gradient was evident among RPs born between 1850 and 1869, followed by RPs born between 1870 and 1884. In the 1885–1900 panel, a social gradient in height was not visible.

**Table 3:** Parental occupational status’s relationship to body height at conscription, OLS.

	1850–1900 (n=3,396)			1850–1869 (n=995)			1870–1884 (n=1,248)			1885–1900 (n=1,153)		
	β	95% CI		β	95% CI		β	95% CI		β	95% CI	
**Parental occupational status**												
Elite	2.82	1.41	4.24	4.52	1.69	7.34	3.37	1.09	5.64	0.63	−1.76	3.01
Middle class	1.59	0.90	2.28	2.31	0.94	3.69	1.57	0.44	2.71	1.18	0.04	2.32
Skilled workers	1.23	0.60	1.85	1.21	−0.03	2.45	1.40	0.39	2.42	1.03	−0.03	2.10
Farmers	1.26	0.55	1.97	2.04	0.67	3.40	1.81	0.65	2.97	−0.11	−1.33	1.11
Unskilled workers	Ref.	Ref.	Ref.	Ref.	Ref.	Ref.	Ref.	Ref.	Ref.	Ref.	Ref.	Ref.
Unknown/no occupation	0.68	−1.83	3.19	1.82	−2.63	6.27	0.93	−3.50	5.35	−2.09	−6.42	2.23
**Birth cohort**												
1850–1869	−1.44	−2.00	−0.87									
1870–1884	Ref.	Ref.	Ref.									
1885–1900	1.73	1.15	2.30									
**Birth year**				0.09	−0.00	0.18	0.13	0.05	0.21	0.03	−0.07	0.14
**Birth region**												
North	0.31	−0.46	1.07	1.78	0.26	3.29	−0.94	−2.21	0.33	1.18	−0.18	2.54
Middle	Ref.	Ref.	Ref.	Ref.	Ref.	Ref.	Ref.	Ref.	Ref.	Ref.	Ref.	Ref.
Coastal	0.45	−0.32	1.21	−0.50	−2.29	1.29	0.43	−0.82	1.68	0.41	−0.80	1.62
South	−1.85	−2.65	−1.06	−0.93	−2.52	0.65	−2.85	−4.16	−1.53	−1.34	−2.70	0.01
Unknown	−0.85	−6.36	4.65	1.42	−8.70	11.55	−4.64	−17.47	8.19	−3.76	−13.05	5.53
**Population size quintile**												
First	0.27	−0.52	1.05	0.32	−1.32	1.96	0.20	−1.03	1.43	0.57	−0.78	1.92
Second	0.01	−0.72	0.75	0.90	−0.51	2.32	−0.29	−1.49	0.91	−0.12	−1.41	1.17
Third	Ref.	Ref.	Ref.	Ref.	Ref.	Ref.	Ref.	Ref.	Ref.	Ref.	Ref.	Ref.
Fourth	0.25	−0.52	1.02	0.36	−1.18	1.89	0.08	−1.17	1.33	0.57	−0.73	1.87
Fifth	−0.39	−1.39	0.60	1.02	−1.26	3.30	−1.38	−2.98	0.22	0.03	−1.73	1.79
**Infant mortality rate in year of birth**												
First	−1.44	−2.22	−0.67	−0.88	−2.57	0.81	−1.15	−2.51	0.22	−1.82	−3.17	−0.47
Second	−0.79	−1.56	−0.03	−0.22	−1.89	1.46	−0.48	−1.77	0.82	−0.79	−2.13	0.54
Third	Ref.	Ref.	Ref.	Ref.	Ref.	Ref.	Ref.	Ref.	Ref.	Ref.	Ref.	Ref.
Fourth	−0.74	−1.51	0.02	0.50	−1.36	2.35	−0.73	−1.94	0.48	−0.45	−2.21	1.30
Fifth	−0.73	−1.52	0.07	0.52	−1.24	2.27	−0.44	−1.70	0.82	−1.46	−3.08	0.17
Unknown	−3.59	−8.58	1.39	2.61	−11.66	16.88	−2.59	−20.68	15.51	−4.19	−9.96	1.58
**Religion**												
Catholic	0.17	−0.45	0.80	0.73	−0.55	2.01	−0.25	−1.25	0.76	−0.02	−1.06	1.01
Liberal Protestant	Ref.	Ref.	Ref.	Ref.	Ref.	Ref.	Ref.	Ref.	Ref.	Ref.	Ref.	Ref.
Neo-Calvinist	−0.08	−0.96	0.80	1.59	−0.27	3.45	−1.32	−2.71	0.07	−0.03	−1.47	1.41
Jewish	−5.70	−7.18	−4.23	−4.85	−8.27	−1.43	−6.06	−8.30	−3.82	−6.25	−8.59	−3.91
No religion	0.64	−0.27	1.55	0.53	−1.32	2.38	−0.58	−2.06	0.90	1.83	0.32	3.33
**Number of siblings**												
Only child	2.13	0.45	3.80	2.59	−0.41	5.58	1.21	−1.47	3.90	3.29	0.03	6.54
One sibling	1.54	0.57	2.52	1.43	−0.44	3.29	0.85	−0.64	2.34	2.95	1.15	4.76
2-4 siblings	Ref.	Ref.	Ref.	Ref.	Ref.	Ref.	Ref.	Ref.	Ref.	Ref.	Ref.	Ref.
5-7 siblings	−0.34	−0.86	0.19	−0.40	−1.43	0.63	−0.20	−1.06	0.65	−0.52	−1.39	0.35
8 or more siblings	−0.84	−1.50	−0.18	−1.78	−3.23	−0.34	−0.54	−1.62	0.53	−0.72	−1.74	0.29
**Parental death?**												
No	Ref.	Ref.	Ref.	Ref.	Ref.	Ref.	Ref.	Ref.	Ref.	Ref.	Ref.	Ref.
Maternal death	−1.03	−1.79	−0.27	−0.95	−2.39	0.49	−0.71	−1.96	0.53	−1.11	−2.41	0.20
Paternal death	0.83	0.11	1.54	1.45	0.03	2.86	1.07	0.00	2.14	0.33	−1.01	1.67
Orphan	−1.96	−3.78	−0.14	−3.40	−6.63	−0.18	0.88	−1.90	3.65	−3.97	−7.79	−0.15
Adjusted R-squared	0.077			0.038			0.058			0.060		

## 4 Discussion

To date, there is no consensus whether social gradients in health was present in historical populations (e.g. Bengtsson et al., 2020; Schenk & van Poppel, 2011). This may be because existing studies on the social gradient in health generally only examined social gradients in mortality. Using other measures of health might help to more fully understand whether a social gradient was present in historical populations.

Other indicators of health and/or morbidity are challenging to find in historical contexts, and are often measured indirectly. For instance, Alter and Riley (1989) measured morbidity with the number of sick days taken from work. Medical reports have also been used to estimate infection rates from specific diseases (Benedictow, 1987). On the whole, however, indicators of health and well-being are seldom available, and when available, seldom collected in a systematic and representative way. Aside from mortality, height is perhaps the best and most widely-available measure of health collected prior to the mid-twentieth century.

This study therefore investigated whether a social gradient in health was present in a historical population, using age at death and body height as measures of health. A sample of Dutch men born between 1850 and 1900 was used. We found no evidence of a social gradient in mortality during this research period. However, being from a farming background was associated with a lower hazard of death, relative to being from an skilled worker background.

There was also some evidence of an occupational gradient in height. This study found a 2.82 cm difference in height between elites and unskilled workers in the full sample panel. This gradient was larger at the beginning of the research period, from 1850 to 1869, and smaller or not present in subsequent cohorts. While there was a general increase in height over the second half of the nineteenth century, this seems to be because of increases in height among low SES individuals (see Fig. 3). It appears that this group benefitted the most from improving environmental conditions (e.g. sanitation improvements, and a more reliable food supply).

This finding broadly aligns with existing research. Among Dutch conscripts born between 1944 and 1947, Huang et al. (2015) found a 1.03 cm gradient based on parental occupational class in height (although they found a gradient of 4.15 cm when using research persons’ educational level instead of parental occupational class). Likewise, today in the Netherlands, the difference in height between similarly-aged men with university and primary education is around 2 cm (Statistics Netherlands, 2008). There appears to be a persistent but small social gradient in height, based on parental occupational class.

If indeed there was a social gradient in health, why might we not have found it in mortality in the nineteenth and early twentieth century Netherlands? This is perhaps because the drivers of height and mortality, while broadly similar, differed somewhat, and represented slightly different facets of health (Alter, 2004). If height and mortality were driven by identical underlying factors, we would expect them to trend more closely together. However, the Dutch secular growth trend in height began to accelerate in the mid-nineteenth century, while the mortality decline began around 1880 (Wolleswinkel van den Bosch et al., 2000). We therefore looked to different explanations for our findings on mortality and height.

Considering causes of death may help to explain the absence of a social gradient in mortality. During this study’s research period, infectious diseases caused a much larger share of deaths than they did after the epidemiological transition, from the mid-twentieth century onward (Wolleswinkel-van den Bosch et al., 2000). With acute infectious diseases, such as smallpox and typhus, underlying health status may have been less relevant for survival than with chronic non-infectious and endemic infectious diseases (McKeown et al., 1972). Whether or not someone was healthy prior to an acute infection was perhaps less important to survival than was being infected in the first place (McKeown et al., 1972). Rather, the proximity to other infected people may have been more important. Evidence for this mortality pattern has been found with tuberculosis (Bengtsson & van Poppel, 2011) and smallpox (Muurling et al., 2022). Once the cause-specific pattern of mortality shifted to a greater share of non-infectious disease deaths, or milder infectious diseases, we perhaps would be able to see a clearer social gradient in mortality emerge.

In this study, we found evidence for the importance of exposure to infectious diseases in determining mortality outcomes in relation to farmers. HISCLASS5’s classification of farmers includes both farm hands and landowners. Both very poor and very rich individuals are included in this group. However, the sons of farmers would have grown up in less population-dense areas, where disease transmission was lower.

Interestingly, we did not find that farmers were taller than other socio-economic groups. This may simply have been because of the socio-economic heterogeneity in this group. Still, farmers, their children, and other rural residents were generally in closer proximity to food sources, and were less vulnerable to fluctuations in food prices. It seems plausible that farmers and others from rural areas would have been taller than peers from higher socio-economic backgrounds. This was indeed the case for the early nineteenth century Netherlands (Tassenaar, 2019). However, from the mid-nineteenth century onward, the Netherlands underwent a shift in its food system, with improvements in production, conservation and transportation (Tassenaar, 2019). The availability and quality of food therefore became more evenly and reliably distributed throughout the Netherlands. Perhaps as a result, heights were increasingly similar across urban and rural Dutch provinces (Tassenaar, 2019). Access to food was increasingly related to the ability to pay for it, rather than to proximity to it (Tassenaar, 2019). This perhaps explains the clearer socio-economic gradient found with height than in mortality.

Taken together, these findings suggest that the social gradient in health may be visible at different points in time, depending on the measure of health used. This finding broadly aligns with Clouston et al. (2016). Inequalities may indeed be a constant feature of society, but the way in which inequalities in disease impact different health outcomes depends heavily on context. As contextual factors – particularly those related to public health improvements – change, so too may the social gradient in health.

### 4.1 Limitations

This study has several limitations that should be taken into account. First, and perhaps most importantly, this study does not exploit cause-specific mortality information. The social gradient in mortality has been found to differ based on cause of death (Debiasi & Dribe, 2020). In the nineteenth- and early twentieth-century Netherlands, municipalities collected cause of death information (in the form of death notes) alongside death certificates. However, with several notable exceptions (Murkens, 2023; Muurling et al., 2022), these death notes have not survived until the present day.

It is also possible that this study’s sample composition obscured a social gradient in mortality. We only included individuals who survived until age twenty. This period saw very high infant and child mortality rates, with the infant mortality rate standing at 211 per 1,000 live births in 1875 (United Nations, 2022). More vulnerable children – perhaps those from lower SES backgrounds – were selectively culled from this study’s sample. Indeed, van Poppel et al. (2005) found evidence of a social gradient, albeit a small one, in child mortality in the nineteenth century Netherlands. Others have similarly found evidence of a gradient in childhood mortality and/or morbidity in other contexts (e.g. Floud et al., 2011; Jaadla et al., 2020; Schneider, 2023). By only examining those who survived until adulthood, it is possible that we understated the social gradient in both mortality and body height.

Additionally, this study to does not consider other factors that are generally found to improve height and mortality, and that may have played a role, such as improvements in sanitation. Sanitation projects have been found to be key drivers of mortality decline (Harris & Helgertz, 2019; Omran, 1971). Such projects were certainly underway in the Netherlands during this study’s research period: this was an important focus of public health efforts in the latter half of the nineteenth century (Quanjer, 2023; Tassenaar, 2014). However, it is not yet known how these efforts impacted health and mortality in the nineteenth and early twentieth century Netherlands. This is currently being investigated by an ongoing project aimed at explaining the mortality transition in Amsterdam (Janssens, 2021). Investigation sanitation’s relationship to both height and mortality in the nineteenth century Netherlands may therefore be possible in the future.

Finally, height is a complex measure of health. Height provides no information on well-being once someone has finished growing. Other indicators, such as weight, would perhaps be better proxies of shorter-term changes in health, although these measures are less commonly available in historical populations (Quanjer & Thompson, 2021; Staub et al., 2010), and were not available for the sample used in this study.

### 4.2 Conclusions

This study examined whether a social gradient in health was present among a sample of Dutch men born in the nineteenth-century Netherlands. Two indicators of health, adult mortality and body height, were studied. We found no clear evidence of a social gradient in adult mortality, but did find some evidence of a social gradient in height between 1850 and 1885. Our study provides evidence that the social gradient in health may be present even when a social gradient in (adult) mortality has not be found. Using multiple measures of health may be important to understand how, why and, perhaps ultimately, when the social gradient in health emerged.

## CRediT statement

Kristina Thompson: Conceptualization, data curation, methodology, formal analysis, visualization, writing – original draft; writing – review & editing.

Johan van Ophem: Conceptualization; methodology, formal analysis, writing – review & editing.

## Ethical statement

The dataset used in this study was derived from anonymized registers, and complies with the.

Dutch Personal Data Protection Act. For further information, please see: https://iisg.amsterdam/en/hsn/privacy-statement.

The authors of this study also declare that they have no competing interests.

## Data Availability

The authors do not have permission to share data.
