# Supplementary material for: Re-examining the social gradient in health: A study of Dutch men, 1850–1984
Source: SSM Popul Health. 2023 Sep 21;24:101518. doi: 10.1016/j.ssmph.2023.101518 (PMC10562747; doi:10.1016/j.ssmph.2023.101518)
Supplement: Multimedia component 1 [file mmc1.docx]

Appendix A. Additional analyses.

Table A.1. Life tables stratified by parental occupational status.

| **Age interval (years)** | **# Alive at start of interval** | **# Deaths** | **# Lost** | **Survival rate** |
| --- | --- | --- | --- | --- |
| **Elite** |  |  |  |  |
| Under 30 | 97 | 7 | 10 | 0.92 |
| 30-45 | 80 | 4 | 7 | 0.88 |
| 46-60 | 68 | 13 | 2 | 0.71 |
| 61-75 | 54 | 24 | 0 | 0.39 |
| 76-90 | 30 | 28 | 0 | 0.03 |
| 91-105 | 2 | 2 | 0 | 0.00 |
| **Middle Class** |  |  |  |  |
| Under 30 | 697 | 38 | 44 | 0.94 |
| 30-45 | 615 | 46 | 21 | 0.87 |
| 46-60 | 548 | 100 | 10 | 0.71 |
| 61-75 | 438 | 185 | 2 | 0.41 |
| 76-90 | 251 | 229 | 0 | 0.04 |
| 91-105 | 22 | 22 | 0 | 0.00 |
| **Skilled Worker** |  |  |  |  |
| Under 30 | 1124 | 73 | 56 | 0.93 |
| 30-45 | 995 | 78 | 34 | 0.86 |
| 45-60 | 883 | 133 | 10 | 0.73 |
| 60-75 | 740 | 333 | 2 | 0.40 |
| 75-90 | 405 | 356 | 1 | 0.05 |
| 90-105 | 48 | 48 | 0 | 0.00 |
| **Farmer** |  |  |  |  |
| Under 30 | 659 | 29 | 26 | 0.96 |
| 30-45 | 604 | 58 | 15 | 0.86 |
| 46-60 | 531 | 73 | 7 | 0.74 |
| 61-75 | 451 | 167 | 0 | 0.47 |
| 76-90 | 284 | 243 | 1 | 0.07 |
| 91-105 | 40 | 40 | 0 | 0.00 |
| **Unskilled Worker** |  |  |  |  |
| Under 30 | 789 | 41 | 45 | 0.95 |
| 30-45 | 703 | 45 | 14 | 0.89 |
| 46-60 | 644 | 102 | 6 | 0.74 |
| 61-75 | 536 | 230 | 3 | 0.42 |
| 76-90 | 393 | 272 | 1 | 0.04 |
| 91-105 | 30 | 30 | 0 | 0.00 |

Table A.2. Parental occupational status’s relationship to age at death, Cox proportional hazards models, stratified by age at last observation.

|  | **Last observation age under age 60**  **(n=1,159; failures=849)** | | | **Last observation age 60 or older**  **(n=2,237; failures=2,227)** | | |
| --- | --- | --- | --- | --- | --- | --- |
|  | HR | 95% CI | | HR | 95% CI | |
| **Parental occupational status** |  |  |  |  |  |  |
| Elite | 1.10 | 0.71 | 1.69 | 1.07 | 0.81 | 1.42 |
| Middle class | 1.11 | 0.90 | 1.37 | 0.99 | 0.87 | 1.12 |
| Skilled workers | 1.07 | 0.88 | 1.30 | 1.01 | 0.89 | 1.13 |
| Farmers | 1.07 | 0.86 | 1.33 | 0.84 | 0.73 | 0.96 |
| Unskilled workers | Ref. | Ref. | Ref. | Ref. | Ref. | Ref. |
| Unknown/no occupation | 1.31 | 0.65 | 2.63 | 0.82 | 0.49 | 1.36 |
| **Birth cohort** |  |  |  |  |  |  |
| 1850-1869 | 1.20 | 1.02 | 1.42 | 1.13 | 1.02 | 1.26 |
| 1870-1884 | Ref. | Ref. | Ref. | Ref. | Ref. | Ref. |
| 1885-1900 | 0.93 | 0.78 | 1.11 | 0.92 | 0.83 | 1.03 |
| **Birth year** |  |  |  |  |  |  |
| **Birth region** |  |  |  |  |  |  |
| North | 0.90 | 0.71 | 1.13 | 0.82 | 0.72 | 0.95 |
| Middle | Ref. | Ref. | Ref. | Ref. | Ref. | Ref. |
| Coastal | 1.19 | 0.95 | 1.49 | 0.82 | 0.71 | 0.94 |
| South | 1.05 | 0.82 | 1.33 | 0.88 | 0.76 | 1.02 |
| Unknown | 1.11 | 0.31 | 4.04 | 1.88 | 0.72 | 4.88 |
| **Population size quintile** |  |  |  |  |  |  |
| First | 0.81 | 0.63 | 1.03 | 1.06 | 0.92 | 1.23 |
| Second | 1.01 | 0.81 | 1.25 | 1.13 | 0.98 | 1.29 |
| Third | Ref. | Ref. | Ref. | Ref. | Ref. | Ref. |
| Fourth | 0.94 | 0.74 | 1.19 | 1.06 | 0.91 | 1.22 |
| Fifth | 0.80 | 0.59 | 1.08 | 1.24 | 1.03 | 1.50 |
| **Infant mortality rate in year of birth** |  |  |  |  |  |  |
| First | 0.99 | 0.78 | 1.26 | 1.03 | 0.89 | 1.19 |
| Second | 1.02 | 0.81 | 1.29 | 1.05 | 0.91 | 1.22 |
| Third | Ref. | Ref. | Ref. | Ref. | Ref. | Ref. |
| Fourth | 0.96 | 0.76 | 1.21 | 0.98 | 0.85 | 1.13 |
| Fifth | 0.97 | 0.77 | 1.23 | 0.97 | 0.84 | 1.13 |
| Unknown | 2.04 | 0.64 | 6.50 | 0.50 | 0.19 | 1.29 |
| **Religion** |  |  |  |  |  |  |
| Catholic | 0.99 | 0.82 | 1.20 | 0.96 | 0.85 | 1.07 |
| Liberal Protestant | Ref. | Ref. | Ref. | Ref. | Ref. | Ref. |
| Neo-Calvinist | 0.91 | 0.69 | 1.20 | 0.94 | 0.80 | 1.11 |
| Jewish | 2.45 | 1.74 | 3.44 | 2.06 | 1.42 | 2.98 |
| No religion | 0.83 | 0.62 | 1.11 | 0.93 | 0.79 | 1.11 |
| **Number of siblings** |  |  |  |  |  |  |
| Only child | 1.50 | 0.94 | 2.41 | 0.92 | 0.64 | 1.32 |
| One sibling | 1.21 | 0.92 | 1.60 | 1.03 | 0.86 | 1.24 |
| 2-4 siblings | Ref. | Ref. | Ref. | Ref. | Ref. | Ref. |
| 5-7 siblings | 1.04 | 0.89 | 1.22 | 0.97 | 0.88 | 1.07 |
| 8 or more siblings | 0.94 | 0.76 | 1.15 | 0.95 | 0.84 | 1.07 |
| **Parental death?** |  |  |  |  |  |  |
| No | Ref. | Ref. | Ref. | Ref. | Ref. | Ref. |
| Maternal death | 1.22 | 0.98 | 1.51 | 0.90 | 0.78 | 1.04 |
| Paternal death | 1.13 | 0.92 | 1.39 | 1.06 | 0.93 | 1.22 |
| Orphan | 0.93 | 0.51 | 1.70 | 0.99 | 0.70 | 1.40 |
| Likelihood-ratio chi^2^ (p-value) | 54.03 | 0.001 |  | 66.21 | 0.000 |  |

Table A.3. RP’s maximum occupational status’s relationship to the hazard of death among RPs over 40, Cox proportional hazards models

|  | **1850-1900**  **(n=2,718;**  **n failures=2,663)** | | | | **1850-1869**  **(n=805;**  **n failures=786)** | | | **1870-1884**  **(n= 1,004;**  **n failures = 982)** | | | **1885-1900**  **(n=909;**  **n failures = 892)** | | |
| --- | --- | --- | --- | --- | --- | --- | --- | --- | --- | --- | --- | --- | --- |
|  | HR | 95% CI | | | HR | 95% CI | | HR | 95% CI | | HR | 95% CI | |
| **RP’s maximum occupational status** |  |  | |  |  |  |  |  |  |  |  |  |  |
| Elite | 1.16 | 0.85 | | 1.59 | 0.95 | 0.56 | 1.59 | 0.94 | 0.48 | 1.85 | 1.83 | 1.10 | 3.06 |
| Middle class | 1.21 | 1.05 | | 1.40 | 1.21 | 0.90 | 1.63 | 1.17 | 0.92 | 1.47 | 1.27 | 0.98 | 1.64 |
| Skilled workers | 1.13 | 1.01 | | 1.27 | 1.21 | 0.98 | 1.50 | 1.07 | 0.88 | 1.29 | 1.21 | 0.99 | 1.47 |
| Farmers | 0.96 | 0.82 | | 1.11 | 0.98 | 0.74 | 1.30 | 0.90 | 0.71 | 1.14 | 1.00 | 0.73 | 1.35 |
| Unskilled workers | Ref. | Ref. | | Ref. | Ref. | Ref. | Ref. | Ref. | Ref. | Ref. | Ref. | Ref. | Ref. |
| Unknown/no occupation | 1.13 | 1.01 | | 1.26 | 1.27 | 1.05 | 1.54 | 1.06 | 0.89 | 1.28 | 1.14 | 0.93 | 1.39 |
| **Birth cohort** |  |  | |  |  |  |  |  |  |  |  |  |  |
| 1850-1869 | 1.16 | 1.05 | | 1.27 |  |  |  |  |  |  |  |  |  |
| 1870-1884 | Ref. | Ref. | | Ref. |  |  |  |  |  |  |  |  |  |
| 1885-1900 | 0.93 | 0.84 | | 1.02 |  |  |  |  |  |  |  |  |  |
| **Birth year** |  |  | |  | 0.98 | 0.97 | 1.00 | 0.99 | 0.98 | 1.01 | 0.99 | 0.97 | 1.00 |
| **Birth region** |  |  | |  |  |  |  |  |  |  |  |  |  |
| North | 0.83 | 0.73 | | 0.94 | 0.83 | 0.65 | 1.05 | 0.85 | 0.68 | 1.05 | 0.86 | 0.68 | 1.10 |
| Middle | Ref. | Ref. | | Ref. | Ref. | Ref. | Ref. | Ref. | Ref. | Ref. | Ref. | Ref. | Ref. |
| Coastal | 0.88 | 0.78 | | 1.00 | 0.89 | 0.68 | 1.17 | 0.82 | 0.66 | 1.01 | 0.98 | 0.79 | 1.22 |
| South | 0.91 | 0.80 | | 1.05 | 0.89 | 0.69 | 1.15 | 0.82 | 0.65 | 1.04 | 1.15 | 0.91 | 1.46 |
| Unknown | 1.59 | 0.69 | | 3.67 | 0.88 | 0.21 | 3.64 | 3.25 | 0.44 | 23.73 | 1.98 | 0.42 | 9.36 |
| **Population size quintile** |  |  | |  |  |  |  |  |  |  |  |  |  |
| First | 1.02 | 0.90 | | 1.17 | 1.09 | 0.84 | 1.41 | 0.98 | 0.80 | 1.21 | 1.11 | 0.86 | 1.42 |
| Second | 1.11 | 0.98 | | 1.25 | 1.24 | 0.99 | 1.55 | 1.06 | 0.86 | 1.31 | 1.14 | 0.89 | 1.45 |
| Third | Ref. | Ref. | | Ref. | Ref. | Ref. | Ref. | Ref. | Ref. | Ref. | Ref. | Ref. | Ref. |
| Fourth | 1.03 | 0.90 | | 1.17 | 0.97 | 0.76 | 1.23 | 0.93 | 0.74 | 1.16 | 1.23 | 0.96 | 1.57 |
| Fifth | 1.10 | 0.93 | | 1.30 | 1.35 | 0.94 | 1.92 | 0.94 | 0.72 | 1.23 | 1.17 | 0.85 | 1.62 |
| **Infant mortality rate in year of birth** |  |  | |  |  |  |  |  |  |  |  |  |  |
| First | 0.98 | 0.86 | | 1.12 | 0.92 | 0.70 | 1.20 | 0.96 | 0.76 | 1.22 | 1.00 | 0.78 | 1.28 |
| Second | 1.04 | 0.91 | | 1.18 | 0.84 | 0.65 | 1.10 | 1.07 | 0.85 | 1.35 | 1.15 | 0.90 | 1.46 |
| Third | Ref. | Ref. | | Ref. | Ref. | Ref. | Ref. | Ref. | Ref. | Ref. | Ref. | Ref. | Ref. |
| Fourth | 0.96 | 0.85 | | 1.10 | 0.81 | 0.60 | 1.09 | 1.11 | 0.90 | 1.38 | 0.86 | 0.63 | 1.18 |
| Fifth | 0.96 | 0.84 | | 1.10 | 0.79 | 0.59 | 1.04 | 1.14 | 0.91 | 1.42 | 0.77 | 0.57 | 1.05 |
| Unknown | 0.74 | 0.34 | | 1.62 |  |  |  | 2.50 | 0.15 | 41.16 | 0.70 | 0.25 | 1.98 |
| **Religion** |  |  | |  |  |  |  |  |  |  |  |  |  |
| Catholic | 0.93 | 0.84 | | 1.04 | 1.01 | 0.83 | 1.24 | 1.04 | 0.87 | 1.25 | 0.79 | 0.66 | 0.95 |
| Liberal Protestant | Ref. | Ref. | | Ref. | Ref. | Ref. | Ref. | Ref. | Ref. | Ref. | Ref. | Ref. | Ref. |
| Neo-Calvinist | 0.92 | 0.80 | | 1.07 | 0.88 | 0.66 | 1.18 | 0.84 | 0.66 | 1.06 | 1.01 | 0.78 | 1.32 |
| Jewish | 2.46 | 1.85 | | 3.27 | 1.78 | 0.90 | 3.54 | 2.43 | 1.51 | 3.90 | 3.59 | 2.26 | 5.68 |
| No religion | 0.88 | 0.76 | | 1.03 | 0.82 | 0.61 | 1.09 | 0.85 | 0.66 | 1.11 | 0.96 | 0.73 | 1.28 |
| **Number of siblings** |  |  | |  |  |  |  |  |  |  |  |  |  |
| Only child | 0.88 | 0.64 | | 1.22 | 1.06 | 0.60 | 1.87 | 0.78 | 0.45 | 1.33 | 0.79 | 0.43 | 1.44 |
| One sibling | 1.02 | 0.87 | | 1.21 | 1.04 | 0.76 | 1.41 | 1.09 | 0.84 | 1.42 | 0.93 | 0.67 | 1.30 |
| 2-4 siblings | Ref. | Ref. | | Ref. | Ref. | Ref. | Ref. | Ref. | Ref. | Ref. | Ref. | Ref. | Ref. |
| 5-7 siblings | 0.97 | 0.89 | | 1.06 | 1.07 | 0.91 | 1.27 | 0.94 | 0.81 | 1.09 | 0.92 | 0.78 | 1.08 |
| 8 or more siblings | 0.92 | 0.82 | | 1.03 | 0.98 | 0.78 | 1.23 | 0.84 | 0.69 | 1.01 | 1.01 | 0.84 | 1.22 |
| **Parental death?** |  |  | |  |  |  |  |  |  |  |  |  |  |
| No | Ref. | Ref. | | Ref. | Ref. | Ref. | Ref. | Ref. | Ref. | Ref. | Ref. | Ref. | Ref. |
| Maternal death | 0.99 | 0.87 | | 1.13 | 0.77 | 0.61 | 0.97 | 1.11 | 0.88 | 1.39 | 1.14 | 0.90 | 1.44 |
| Paternal death | 1.07 | 0.94 | | 1.21 | 1.09 | 0.87 | 1.38 | 0.97 | 0.80 | 1.17 | 1.20 | 0.94 | 1.54 |
| Orphan | 0.93 | 0.67 | | 1.29 | 0.75 | 0.41 | 1.38 | 1.31 | 0.80 | 2.14 | 0.86 | 0.44 | 1.68 |
| Likelihood-ratio chi^2^ (p-value) | 93.20 | 0.000 | |  | 42.79 | 0.048 |  | 38.11 | 0.147 |  | 67.59 | 0.000 |  |
|  | | |  |  |  |  |  |  |  |  |  |  |  |

Table A.4. Parental occupational status (HISCAM)’s relationship to age at death, Cox proportional hazards models

|  | **1850-1900**  **(n=3,034;**  **n failures=2,751)** | | | **1850-1869**  **(n=860;**  **n failures = 794))** | | | **1870-1884**  **(n=1,107;**  **n failures = 1,006)** | | | **1885-1900**  **(n=1,067;**  **n failures = 951)** | | |
| --- | --- | --- | --- | --- | --- | --- | --- | --- | --- | --- | --- | --- |
|  | HR | 95% CI | | HR | 95% CI | | HR | 95% CI | | HR | 95% CI | |
| **Parental occupational status (HISCAM)** | 1.00 | 1.00 | 1.00 | 1.00 | 0.99 | 1.01 | 1.00 | 0.99 | 1.01 | 1.00 | 0.99 | 1.00 |
| **Birth cohort** |  |  |  |  |  |  |  |  |  |  |  |  |
| 1850-1869 | 1.17 | 1.06 | 1.29 |  |  |  |  |  |  |  |  |  |
| 1870-1884 | Ref. | Ref. | Ref. |  |  |  |  |  |  |  |  |  |
| 1885-1900 | 0.93 | 0.84 | 1.02 |  |  |  |  |  |  |  |  |  |
| **Birth year** |  |  |  | 0.98 | 0.97 | 1.00 | 0.99 | 0.97 | 1.00 | 0.98 | 0.96 | 1.00 |
| **Birth region** |  |  |  |  |  |  |  |  |  |  |  |  |
| North | 0.85 | 0.75 | 0.96 | 0.88 | 0.70 | 1.11 | 0.85 | 0.69 | 1.06 | 0.85 | 0.68 | 1.07 |
| Middle | Ref. | Ref. | Ref. | Ref. | Ref. | Ref. | Ref. | Ref. | Ref. | Ref. | Ref. | Ref. |
| Coastal | 0.91 | 0.80 | 1.04 | 0.93 | 0.70 | 1.24 | 0.88 | 0.71 | 1.09 | 0.95 | 0.77 | 1.17 |
| South | 0.93 | 0.81 | 1.06 | 0.94 | 0.73 | 1.20 | 0.85 | 0.67 | 1.07 | 1.15 | 0.91 | 1.44 |
| Unknown | 1.70 | 0.74 | 3.92 | 0.72 | 0.10 | 5.27 | 2.43 | 0.33 | 17.63 | 1.92 | 0.37 | 9.90 |
| **Population size quintile** |  |  |  |  |  |  |  |  |  |  |  |  |
| First | 1.01 | 0.89 | 1.15 | 1.03 | 0.79 | 1.32 | 1.04 | 0.84 | 1.28 | 1.03 | 0.81 | 1.30 |
| Second | 1.09 | 0.97 | 1.24 | 1.13 | 0.91 | 1.41 | 1.14 | 0.93 | 1.40 | 1.12 | 0.89 | 1.40 |
| Third | Ref. | Ref. | Ref. | Ref. | Ref. | Ref. | Ref. | Ref. | Ref. | Ref. | Ref. | Ref. |
| Fourth | 1.05 | 0.93 | 1.20 | 0.94 | 0.74 | 1.18 | 0.97 | 0.77 | 1.21 | 1.29 | 1.02 | 1.63 |
| Fifth | 1.12 | 0.94 | 1.32 | 1.16 | 0.80 | 1.67 | 1.00 | 0.77 | 1.32 | 1.31 | 0.94 | 1.82 |
| **Infant mortality rate in year of birth** |  |  |  |  |  |  |  |  |  |  |  |  |
| First | 1.01 | 0.89 | 1.15 | 0.88 | 0.68 | 1.15 | 0.98 | 0.77 | 1.23 | 1.09 | 0.85 | 1.39 |
| Second | 1.05 | 0.92 | 1.19 | 0.80 | 0.61 | 1.04 | 1.10 | 0.87 | 1.38 | 1.20 | 0.94 | 1.53 |
| Third | Ref. | Ref. | Ref. | Ref. | Ref. | Ref. | Ref. | Ref. | Ref. | Ref. | Ref. | Ref. |
| Fourth | 1.00 | 0.88 | 1.13 | 0.84 | 0.63 | 1.14 | 1.16 | 0.94 | 1.44 | 0.86 | 0.63 | 1.17 |
| Fifth | 0.97 | 0.85 | 1.11 | 0.82 | 0.62 | 1.09 | 1.17 | 0.94 | 1.46 | 0.83 | 0.61 | 1.12 |
| Unknown | 0.83 | 0.38 | 1.81 | 11.29 | 0.97 | 131.95 | 1.66 | 0.10 | 26.97 | 0.64 | 0.20 | 2.08 |
| **Religion** |  |  |  |  |  |  |  |  |  |  |  |  |
| Catholic | 0.94 | 0.85 | 1.05 | 1.00 | 0.82 | 1.23 | 1.03 | 0.86 | 1.23 | 0.83 | 0.69 | 0.98 |
| Liberal Protestant | Ref. | Ref. | Ref. | Ref. | Ref. | Ref. | Ref. | Ref. | Ref. | Ref. | Ref. | Ref. |
| Neo-Calvinist | 0.91 | 0.79 | 1.05 | 0.76 | 0.56 | 1.02 | 0.86 | 0.68 | 1.09 | 1.10 | 0.86 | 1.42 |
| Jewish | 2.34 | 1.80 | 3.03 | 2.06 | 1.17 | 3.61 | 2.48 | 1.62 | 3.81 | 2.87 | 1.88 | 4.39 |
| No religion | 0.91 | 0.78 | 1.06 | 0.85 | 0.63 | 1.13 | 0.85 | 0.66 | 1.10 | 1.01 | 0.76 | 1.33 |
| **Number of siblings** |  |  |  |  |  |  |  |  |  |  |  |  |
| Only child | 1.03 | 0.74 | 1.43 | 1.37 | 0.78 | 2.41 | 1.01 | 0.60 | 1.70 | 0.77 | 0.39 | 1.50 |
| One sibling | 1.05 | 0.89 | 1.23 | 1.18 | 0.88 | 1.58 | 1.08 | 0.83 | 1.40 | 0.93 | 0.67 | 1.28 |
| 2-4 siblings | Ref. | Ref. | Ref. | Ref. | Ref. | Ref. | Ref. | Ref. | Ref. | Ref. | Ref. | Ref. |
| 5-7 siblings | 0.99 | 0.91 | 1.08 | 1.16 | 0.98 | 1.36 | 0.95 | 0.82 | 1.10 | 0.92 | 0.79 | 1.07 |
| 8 or more siblings | 0.95 | 0.86 | 1.06 | 1.02 | 0.82 | 1.27 | 0.87 | 0.72 | 1.04 | 1.04 | 0.87 | 1.24 |
| **Parental death?** |  |  |  |  |  |  |  |  |  |  |  |  |
| No | Ref. | Ref. | Ref. | Ref. | Ref. | Ref. | Ref. | Ref. | Ref. | Ref. | Ref. | Ref. |
| Maternal death | 1.01 | 0.89 | 1.15 | 0.80 | 0.64 | 1.01 | 1.25 | 1.01 | 1.55 | 1.04 | 0.83 | 1.30 |
| Paternal death | 1.05 | 0.93 | 1.18 | 1.10 | 0.88 | 1.38 | 0.94 | 0.78 | 1.13 | 1.20 | 0.95 | 1.52 |
| Orphan | 0.93 | 0.68 | 1.27 | 0.90 | 0.53 | 1.51 | 1.21 | 0.74 | 1.98 | 0.79 | 0.41 | 1.54 |
| Likelihood-ratio chi^2^ (p-value) | 78.06 | 0.000 |  | 36.80 | 0.078 |  | 37.72 | 0.064 |  | 55.96 | 0.000 |  |

Table A.5. Parental occupational status (HISCAM)’s relationship to height, OLS

|  | **1850-1900**  **(n=3,034)** | | | **1850-1869**  **(n=860)** | | | **1870-1884**  **(n=1,107)** | | | **1885-1900**  **(n=1,067)** | | |
| --- | --- | --- | --- | --- | --- | --- | --- | --- | --- | --- | --- | --- |
|  | β | 95% CI | | β | 95% CI | | β | 95% CI | | β | 95% CI | |
| **Parental occupational status (HISCAM)** | 0.04 | 0.01 | 0.07 | 0.07 | 0.01 | 0.15 | 0.07 | 0.02 | 0.12 | 0.01 | -0.02 | 0.05 |
| **Birth cohort** |  |  |  |  |  |  |  |  |  |  |  |  |
| 1850-1869 | -1.53 | -2.13 | -0.92 |  |  |  |  |  |  |  |  |  |
| 1870-1884 | Ref. | Ref. | Ref. |  |  |  |  |  |  |  |  |  |
| 1885-1900 | 1.67 | 1.07 | 2.27 |  |  |  |  |  |  |  |  |  |
| **Birth year** |  |  |  | 0.07 | -0.03 | 0.17 | 0.14 | 0.05 | 0.22 | 0.00 | -0.11 | 0.11 |
| **Birth region** |  |  |  |  |  |  |  |  |  |  |  |  |
| North | 0.19 | -0.62 | 1.00 | 1.05 | -0.54 | 2.64 | -0.75 | -2.12 | 0.62 | 0.99 | -0.40 | 2.38 |
| Middle | Ref. | Ref. | Ref. | Ref. | Ref. | Ref. | Ref. | Ref. | Ref. | Ref. | Ref. | Ref. |
| Coastal | 0.37 | -0.45 | 1.18 | -0.72 | -2.67 | 1.23 | 0.43 | -0.91 | 1.76 | 0.22 | -1.06 | 1.49 |
| South | -2.00 | -2.84 | -1.15 | -1.25 | -2.94 | 0.43 | -2.88 | -4.29 | -1.47 | -1.56 | -2.98 | -0.14 |
| Unknown | -3.78 | -10.05 | 2.49 | -8.32 | -22.53 | 5.88 | -4.90 | -17.80 | 7.99 | -4.66 | -14.39 | 5.07 |
| **Population size quintile** |  |  |  |  |  |  |  |  |  |  |  |  |
| First | 0.32 | -0.49 | 1.14 | 0.67 | -1.05 | 2.40 | 0.12 | -1.18 | 1.43 | 0.21 | -1.16 | 1.58 |
| Second | 0.19 | -0.59 | 0.96 | 1.35 | -0.15 | 2.85 | -0.31 | -1.59 | 0.96 | -0.08 | -1.41 | 1.25 |
| Third | Ref. | Ref. | Ref. | Ref. | Ref. | Ref. | Ref. | Ref. | Ref. | Ref. | Ref. | Ref. |
| Fourth | 0.15 | -0.66 | 0.97 | -0.14 | -1.75 | 1.47 | -0.15 | -1.49 | 1.20 | 0.75 | -0.61 | 2.11 |
| Fifth | -0.43 | -1.50 | 0.64 | 1.31 | -1.16 | 3.79 | -1.81 | -3.52 | -0.10 | 0.01 | -1.88 | 1.90 |
| **Infant mortality rate in year of birth** |  |  |  |  |  |  |  |  |  |  |  |  |
| First | -1.35 | -2.17 | -0.53 | -1.03 | -2.82 | 0.77 | -0.67 | -2.11 | 0.77 | -2.13 | -3.56 | -0.70 |
| Second | -0.83 | -1.64 | -0.01 | -0.94 | -2.76 | 0.88 | 0.07 | -1.32 | 1.47 | -1.18 | -2.59 | 0.23 |
| Third | Ref. | Ref. | Ref. | Ref. | Ref. | Ref. | Ref. | Ref. | Ref. | Ref. | Ref. | Ref. |
| Fourth | -0.64 | -1.45 | 0.18 | -0.25 | -2.27 | 1.76 | -0.01 | -1.32 | 1.30 | -0.68 | -2.52 | 1.17 |
| Fifth | -0.66 | -1.51 | 0.19 | 0.11 | -1.80 | 2.03 | 0.05 | -1.29 | 1.40 | -2.02 | -3.75 | -0.28 |
| Unknown | -1.80 | -7.42 | 3.82 | 11.40 | -6.14 | 28.94 | -1.25 | -19.42 | 16.92 | -4.06 | -10.51 | 2.40 |
| **Religion** |  |  |  |  |  |  |  |  |  |  |  |  |
| Catholic | 0.31 | -0.35 | 0.97 | 0.42 | -0.95 | 1.79 | 0.14 | -0.94 | 1.22 | 0.24 | -0.83 | 1.32 |
| Liberal Protestant | Ref. | Ref. | Ref. | Ref. | Ref. | Ref. | Ref. | Ref. | Ref. | Ref. | Ref. | Ref. |
| Neo-Calvinist | -0.28 | -1.20 | 0.65 | 0.49 | -1.55 | 2.53 | -0.94 | -2.41 | 0.53 | -0.07 | -1.57 | 1.42 |
| Jewish | -5.34 | -6.95 | -3.73 | -4.81 | -8.65 | -0.98 | -5.51 | -8.05 | -2.98 | -6.01 | -8.45 | -3.57 |
| No religion | 0.68 | -0.29 | 1.66 | 0.46 | -1.55 | 2.46 | -0.18 | -1.78 | 1.42 | 1.46 | -0.13 | 3.06 |
| **Number of siblings** |  |  |  |  |  |  |  |  |  |  |  |  |
| Only child | 1.39 | -0.52 | 3.31 | 1.78 | -1.75 | 5.31 | 0.58 | -2.45 | 3.61 | 2.34 | -1.23 | 5.91 |
| One sibling | 1.50 | 0.47 | 2.52 | 1.47 | -0.52 | 3.47 | 0.75 | -0.85 | 2.34 | 2.67 | 0.81 | 4.52 |
| 2-4 siblings | Ref. | Ref. | Ref. | Ref. | Ref. | Ref. | Ref. | Ref. | Ref. | Ref. | Ref. | Ref. |
| 5-7 siblings | -0.43 | -0.98 | 0.13 | -0.53 | -1.64 | 0.58 | -0.24 | -1.15 | 0.67 | -0.56 | -1.47 | 0.36 |
| 8 or more siblings | -0.87 | -1.56 | -0.18 | -2.11 | -3.63 | -0.60 | -0.30 | -1.44 | 0.84 | -0.74 | -1.79 | 0.32 |
| **Parental death?** |  |  |  |  |  |  |  |  |  |  |  |  |
| No | Ref. | Ref. | Ref. | Ref. | Ref. | Ref. | Ref. | Ref. | Ref. | Ref. | Ref. | Ref. |
| Maternal death | -1.28 | -2.08 | -0.48 | -1.37 | -2.93 | 0.18 | -1.12 | -2.44 | 0.20 | -1.08 | -2.42 | 0.27 |
| Paternal death | 0.94 | 0.18 | 1.70 | 1.49 | -0.03 | 3.02 | 1.22 | 0.09 | 2.34 | 0.38 | -1.03 | 1.80 |
| Orphan | -2.17 | -4.06 | -0.27 | -3.15 | -6.46 | 0.15 | 0.44 | -2.56 | 3.44 | -3.63 | -7.47 | 0.20 |
| Adjusted R^2^ | 0.073 |  |  | 0.027 |  |  | 0.052 |  |  | 0.052 |  |  |

Table A.6 Height’s relationship to mortality, Cox proportional hazards models.

|  | **1850-1900**  **(n=3,396;**  **failures/deaths=3,076)** | | | **1850-1869**  **(n=995;**  **failures/deaths=920)** | | | **1870-1885**  **(n=1,248;**  **failures/deaths=1,130)** | | | **1885-1900**  **(n=1,153;**  **failures/deaths= 1,026** | | |
| --- | --- | --- | --- | --- | --- | --- | --- | --- | --- | --- | --- | --- |
|  | HR | 95% CI | | HR | 95% CI | | HR | 95% CI | | HR | 95% CI | |
| **Body height (cm)** | 0.85 | 0.73 | 0.98 | 0.90 | 0.71 | 1.15 | 0.73 | 0.57 | 0.95 | 0.76 | 0.54 | 1.05 |
| **Body height (cm)^2^** | 1.00 | 1.00 | 1.00 | 1.00 | 1.00 | 1.00 | 1.00 | 1.00 | 1.00 | 1.00 | 1.00 | 1.00 |
| **Birth cohort** |  |  |  |  |  |  |  |  |  |  |  |  |
| 1850-1869 | 1.15 | 1.05 | 1.26 |  |  |  |  |  |  |  |  |  |
| 1870-1884 | Ref. | Ref. | Ref. |  |  |  |  |  |  |  |  |  |
| 1885-1900 | 0.92 | 0.84 | 1.01 |  |  |  |  |  |  |  |  |  |
| **Birth year** |  |  |  | 0.98 | 0.97 | 1.00 | 0.99 | 0.98 | 1.00 | 0.98 | 0.96 | 1.00 |
| **Birth region** |  |  |  |  |  |  |  |  |  |  |  |  |
| North | 0.85 | 0.76 | 0.96 | 0.91 | 0.73 | 1.13 | 0.80 | 0.66 | 0.98 | 0.86 | 0.69 | 1.08 |
| Middle | Ref. | Ref. | Ref. | Ref. | Ref. | Ref. | Ref. | Ref. | Ref. | Ref. | Ref. | Ref. |
| Coastal | 0.91 | 0.81 | 1.02 | 0.92 | 0.71 | 1.19 | 0.83 | 0.68 | 1.01 | 0.98 | 0.80 | 1.19 |
| South | 0.94 | 0.83 | 1.06 | 0.96 | 0.76 | 1.22 | 0.78 | 0.63 | 0.98 | 1.19 | 0.96 | 1.49 |
| Unknown | 1.72 | 0.81 | 3.64 | 0.90 | 0.22 | 3.69 | 2.27 | 0.31 | 16.45 | 1.98 | 0.43 | 9.24 |
| **Population size quintile** |  |  |  |  |  |  |  |  |  |  |  |  |
| First | 0.98 | 0.87 | 1.11 | 1.06 | 0.83 | 1.34 | 0.94 | 0.77 | 1.14 | 1.00 | 0.80 | 1.26 |
| Second | 1.09 | 0.97 | 1.22 | 1.19 | 0.97 | 1.46 | 1.05 | 0.87 | 1.28 | 1.11 | 0.89 | 1.38 |
| Third | Ref. | Ref. | Ref. | Ref. | Ref. | Ref. | Ref. | Ref. | Ref. | Ref. | Ref. | Ref. |
| Fourth | 1.04 | 0.92 | 1.17 | 0.98 | 0.79 | 1.23 | 0.94 | 0.77 | 1.16 | 1.23 | 0.98 | 1.54 |
| Fifth | 1.12 | 0.96 | 1.31 | 1.38 | 0.99 | 1.92 | 0.94 | 0.73 | 1.21 | 1.21 | 0.90 | 1.64 |
| **Infant mortality rate in year of birth** |  |  |  |  |  |  |  |  |  |  |  |  |
| First | 0.99 | 0.88 | 1.12 | 0.92 | 0.72 | 1.18 | 0.91 | 0.74 | 1.14 | 1.05 | 0.84 | 1.32 |
| Second | 1.03 | 0.91 | 1.16 | 0.81 | 0.63 | 1.04 | 1.05 | 0.85 | 1.31 | 1.20 | 0.96 | 1.50 |
| Third | Ref. | Ref. | Ref. | Ref. | Ref. | Ref. | Ref. | Ref. | Ref. | Ref. | Ref. | Ref. |
| Fourth | 0.98 | 0.87 | 1.10 | 0.83 | 0.63 | 1.09 | 1.10 | 0.90 | 1.34 | 0.86 | 0.64 | 1.16 |
| Fifth | 0.97 | 0.85 | 1.09 | 0.82 | 0.63 | 1.07 | 1.08 | 0.87 | 1.32 | 0.83 | 0.62 | 1.10 |
| Unknown | 0.78 | 0.38 | 1.58 | 8.25 | 1.11 | 61.10 | 1.64 | 0.10 | 26.62 | 0.64 | 0.23 | 1.77 |
| **Religion** |  |  |  |  |  |  |  |  |  |  |  |  |
| Catholic | 0.96 | 0.87 | 1.06 | 1.06 | 0.88 | 1.28 | 1.05 | 0.89 | 1.24 | 0.83 | 0.70 | 0.98 |
| Liberal Protestant | Ref. | Ref. | Ref. | Ref. | Ref. | Ref. | Ref. | Ref. | Ref. | Ref. | Ref. | Ref. |
| Neo-Calvinist | 0.94 | 0.82 | 1.08 | 0.88 | 0.67 | 1.16 | 0.85 | 0.68 | 1.06 | 1.07 | 0.84 | 1.37 |
| Jewish | 2.20 | 1.72 | 2.82 | 1.61 | 0.95 | 2.73 | 2.20 | 1.48 | 3.27 | 2.99 | 1.99 | 4.52 |
| No religion | 0.89 | 0.77 | 1.03 | 0.83 | 0.64 | 1.09 | 0.86 | 0.68 | 1.09 | 1.00 | 0.77 | 1.31 |
| **Number of siblings** |  |  |  |  |  |  |  |  |  |  |  |  |
| Only child | 1.03 | 0.79 | 1.36 | 1.37 | 0.86 | 2.17 | 1.10 | 0.70 | 1.72 | 0.71 | 0.41 | 1.22 |
| One sibling | 1.07 | 0.91 | 1.24 | 1.18 | 0.90 | 1.55 | 1.12 | 0.88 | 1.43 | 0.86 | 0.63 | 1.19 |
| 2-4 siblings | Ref. | Ref. | Ref. | Ref. | Ref. | Ref. | Ref. | Ref. | Ref. | Ref. | Ref. | Ref. |
| 5-7 siblings | 0.99 | 0.91 | 1.07 | 1.13 | 0.97 | 1.32 | 0.95 | 0.83 | 1.09 | 0.91 | 0.79 | 1.06 |
| 8 or more siblings | 0.94 | 0.85 | 1.04 | 1.02 | 0.83 | 1.26 | 0.86 | 0.72 | 1.02 | 1.01 | 0.85 | 1.20 |
| **Parental death?** |  |  |  |  |  |  |  |  |  |  |  |  |
| No | Ref. | Ref. | Ref. | Ref. | Ref. | Ref. | Ref. | Ref. | Ref. | Ref. | Ref. | Ref. |
| Maternal death | 0.99 | 0.88 | 1.12 | 0.78 | 0.63 | 0.97 | 1.19 | 0.97 | 1.46 | 1.05 | 0.84 | 1.31 |
| Paternal death | 1.07 | 0.96 | 1.20 | 1.13 | 0.91 | 1.40 | 1.00 | 0.84 | 1.18 | 1.17 | 0.94 | 1.47 |
| Orphan | 1.00 | 0.74 | 1.35 | 0.99 | 0.60 | 1.64 | 1.27 | 0.80 | 2.03 | 0.78 | 0.40 | 1.52 |
| Restricted F-test (chi^2^ statistic and p-value) | 4.86 | 0.027 |  | 0.70 | 0.403 |  | 5.45 | 0.020 |  | 2.96 | 0.086 |  |
